# Index construction and application of digital transformation in the insurance industry: Evidence from China

**DOI:** 10.1371/journal.pone.0296899

**Published:** 2024-01-18

**Authors:** Mengqi Liu, Han Yang, Shuxin Zheng

**Affiliations:** 1 School of Finance, Southwestern University of Finance and Economics, Chengdu, Sichuan, China; 2 College of Economics and Management, Fujian Agriculture and Forestry University, Fuzhou, Fujian, China; Sichuan Agricultural University, CHINA

## Abstract

In the context of digitization, the insurance industry’s value chain is undergoing significant shifts. However, the existing research on its comprehension and measurement remains relatively limited. This study constructs an index system for digital transformation in the insurance industry (DTII) on three components: digital infrastructure, digital platform, and digital applications. Utilizing data from 31 provinces in China, this study employs the entropy weight method, analytic hierarchy process method and minimum relative entropy method to measure the weights of indicators, empirically applying this index system. The results show that DTII in China experiences rapid advancement with an average annual growth rate of 20.46% from 2014 to 2020 and there exists strong regional convergence. In addition, the spatial agglomeration and spatial effects of DTII are mainly concentrated in the life insurance industry and the eastern region. This study provides an index system and empirical evidence for evaluating the DTII, providing policy insights for exploring the sustainable development path of the insurance industry in the digital era.

## Introduction

Digitization stands as an essential and fundamental element of contemporary economic activity, encompassing the process of transforming analog information into digital form and natural language into machine language [1]. With the wide adoption of new digital technologies such as Artificial Intelligence, Block Chain, Big Data, and Cloud Computing, information and data have emerged as pivotal factors driving industrial development [2]. Digital transformation has brought about substantial alterations in the sustainability of industries by reshaping traditional production methods, business models, and innovation approaches by reducing search, marginal, and transaction costs [3].

The insurance industry is a field closely intertwined with data and information, with its theoretical foundation rooted in the law of large numbers and probability theory [4]. The widespread utilization of digital technology is driving a profound transformation of the insurance industry’s value chain, giving rise to emerging digital insurance products and services such as smart contracts and usage-based insurance [2]. However, the current study on the application of digital technologies in the insurance industry and the impact of digital transformation is mainly theoretical [5, 6], with relatively limited comprehension and measurement of digital transformation in the insurance industry (DTII). The existing empirical research focuses on the application of InsurTech and its impact on the traditional insurance industry, mainly emphasizing the changes brought by emerging technologies and innovative business models in the traditional insurance industry [7], such as improving efficiency [8], creating business value chains [9] and enhancing risk management [2], etc.

DTII represents a systematic strategic revolution [10], making precise assessment of its extent exceptionally crucial. In current research on digital transformation, there are various methods for measuring digital transformation. These methods primarily include case analysis, survey research, and textual analysis to measures digital transformation at both industry and firm levels.

Case analysis focuses on the study of digital transformation using a single-case or multiple-case approach. For example, Antonopoulou et al.(2023) conduct a qualitative case study of a higher education institution in the United Kingdom, exploring the integration of digital transformation with organizational change during the COVID-19 pandemic [11]. Although this method can deeply explore the process of digital transformation and the interaction mechanism between various elements [12], it suffers from the problem of strong subjectivity and weak generalizability.

Survey research primarily assesses digital transformation by collecting survey data. Skare et al. (2023) constructed a digital transformation index for small and medium-sized enterprises using the Digital Economy and Society Index from the European Business Financing Survey [13]. While this survey method can reveal the dynamic process of digital transformation, it also faces challenges such as small sample sizes, limited representativeness, and difficulties in conducting panel studies.

In terms of textual analysis, researchers mainly use public platform searches for information related to digital transformation, such as business registration data and annual reports. For example, Cao et al. (2020) measure the application of digital technology in the insurance industry by counting the frequency of digital technology in insurance news reports [14]. While textual analysis offers significant advantages in information extraction and pattern discovery, digital transformation is a systemic and dynamic process, compassing the application of digital technology, product and service enhancements, business model updating, etc. Relying solely on keywords cannot adequately reflect the digital transformation behaviors of industries and companies [15]. Furthermore, the insurance industry differs significantly from the manufacturing industry, which is the primary focus of current research [16]. The insurance industry has unique business and industry structures, making existing methods of measuring digital transformation less suitable for this sector.

Therefore, our research takes the insurance industry as the research object and constructs a comprehensive index system for the digital transformation of the insurance industry. We take China as an example to provide empirical experience in assessing the economic effects of digital transformation in the insurance industry. China, as a developing country with the largest economic volume in the world, its insurance industry is experiencing a rough patch from the extensive growth mode to the refined growth mode [12], which means they need more elaborate transformation to better meet customer needs and create business values thus to acquire continuous development and success under changes in the rapidly developed digital society. Digital transformation becomes a key driver to achieve such industrial transformation [17]. In this context, it is essential to learn and investigate the present status and features of DTII in China. Therefore, China serves as a typical example for us to explore DTII. On the one hand, as the world’s second-largest economy, China’s DTII has an important impact on the global economy and the worldwide insurance market. On the other hand, China’s experience in DTII can provide useful insights for other regions and countries seeking to formulate effective strategies and policies for their own DTII.

This study deconstructs the concept of DTII, and establishes an index system for DTII on three components: digital infrastructure (DI), digital platform (DP), and digital applications (DA). Furthermore, we empirically analyze the development trends and regional characteristics of digital transformation in China’s insurance industry from 2014 to 2020. We utilize the spatial Durbin model (SDM) to investigate the spatial spillover effects of digital transformation on the development of insurance industry.

The original contributions of this study are as follows: First, this study defines the concept of DTII from the perspectives of DI, DP, and DA, thereby offering a new perspective for research on DTII. The existing literature often sporadically views digital transformation as the mere application of digital technologies, neglecting its potential to transform products and services and shape industry-wide digital strategies [17, 18]. Second, our study constructs a comprehensive set of indicators to measure DTII, thus contributing to the enrichment of academic research on DTII. Furthermore, the DTII index system we have developed is universal and can be applied to other industries for evaluating the progress of digital transformation. To date, there is no comprehensive and scientifically devised set of industry-level metrics that encapsulate the current state of DTII in China. Existing literature primarily focuses on measuring the level of digital transformation within insurance companies by utilizing InsurTech [19] or examining the frequency of digital-related keywords in insurers’ annual reports [14]. Third, this paper compiles a regional index of DTII, which reflects the degree of development and regional balance of digital transformation in China’s insurance industry. The index aims to help policymakers and industry practitioners better understand the spatial and temporal characteristics of DTII in China. The current research primarily focuses on digital transformation at the insurance company level [20, 21], with a lack of empirical analysis at the industry and regional levels. Fourth, taking into consideration the potential spatial spillover effects of DTII, we examine the impact of DTII on its development from a spatial spillover perspective, providing a supplementary dimension to the study of the relationship between digital transformation and the insurance industry’s development. Most of the existing studies use panel models [14, 22], ignoring the spatial effects of DTII. Fifth, we comparatively analyze the differences in the impact of DTII on the development of the insurance industry between the life insurance industry and the property insurance industry, as well as the eastern, central and western regions, aiming to provide China’s experience for DTII in other regions and countries, which can promote the international research related to DTII in the future.

## Conceptual model

### The concept of DTII

Digital transformation is one of the latest trends in the global market, and more and more companies and industries are undergoing digital transformation to maintain competitive advantage and sustainability by changing their business processes or creating new business models [10]. Like most other industries, the insurance industry is strongly influenced by digital transformation in terms of updating business models, opening up new channels to attract customers, and creating digital experiences for users [23].

Currently, there is no uniform definition of DTII. Based on the perspective of insurance companies, most literature defines DTII as an insurance company upgrading its organizational structure, business model, and internal processes through a series of technological applications and innovations to improve service quality and efficiency in the context of the digital age [24]. This definition is essentially a customer-centric digital development strategy for insurers [25] and hardly reflects the full picture of DTII as a whole.

We try to systematically reflect the process of DTII. Digital transformation includes not only the adoption of new communication technologies but also an opportunity to digitally reshape products, services and industrial strategies [18]. Thus, our definition of DTII makes further extensions beyond the application of digital technology to the insurance industry and related companies considered by previous scholars. Specifically, we define DTII as a sustainable development process of the insurance industry with DI as the physical basis, DP as the key medium, and DA as the concentrated expression. DI is the necessary prerequisite and material foundation for DTII, which refers to an infrastructure system based on an information network that can integrate new information technologies [26]. DI is the necessary prerequisite and material foundation for DTII. Without the support of DI, it will be difficult for DTII to move forward. DP is a technology architecture that can integrate information, computing, and connectivity technology platforms available to the organization with the developing computing capabilities [27]. As the pivotal vehicle for DTII, DP unlocks more potential for innovative insurance products, businesses, and business models. DA is the utilization of advanced digital technologies that can drive other technologies and generate new business or market and business models [28]. DA is the most direct place for consumers to experience DTII. DA reflects not only the impact of DTII on individuals, insurance companies, the insurance industry, and even society, but also the digitalization process of the insurance industry. This includes the growing diversity consumer needs, insurance companies’ imperatives to enhance operational efficiency and reshape their business models, and the insurance industry’s demands for integrating the upstream and downstream industrial chains.

To sum up, DTII follows an ecosystem with digital characteristics, that is, starting from the underlying DI, relying on the DP in the middle layer to maximize the digital ecological chain of terminal applications represented by DA. DI, DP, and DA interact to form a comprehensive indicator system for DTII, and we describe these three Indicators in the following sections.

Therefore, this study not only clarifies the role and characteristics of DTII, but also introduces a comprehensive and quantitative tool to measure the level of DTII. This tool holds the potential to drive future international research on DTII. It provides valuable insights into the digital transformation of the global insurance industry and provides more guidance for international competition in the insurance industry. Moreover, this research highlights the importance and necessity of digital transformation for the continued development of the insurance industry, thus contributing to global risk management, cooperation and the promotion of international economic stability.

### Constructing a conceptual model

**Fig 1:**
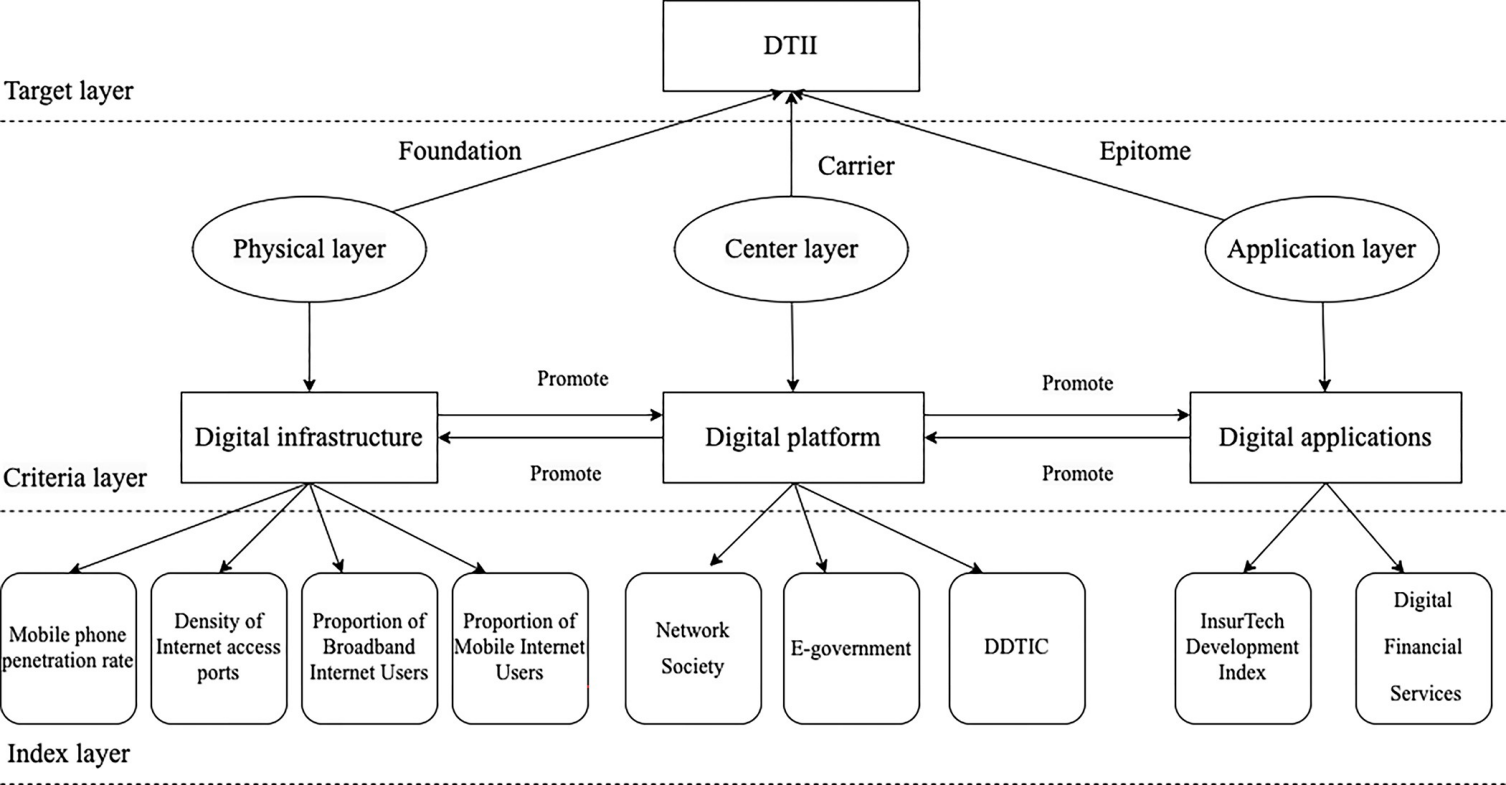
The research framework for constructing DTII index system.

The definition of a conceptual model is the basis for the construction of an evaluation index system. To construct a normative conceptual model, we first define the phenomenon to be measured and its sub-indicators [29]. After defining DTII, we construct an indicator system for DTII (Fig 1), which is composed of DI, DP, and DA. We describe and analyze the definition and measurement of each indicator in this chapter.

### DI

DI is an infrastructure system based on an information network and integrating new generations of information technology [26]. Existing literature links DI to various favorable socioeconomic outcomes [30]. At the individual level, DI development is associated with increased income [31], poverty reduction [32], and incentives for entrepreneurial behavior [33]. At the firm level, the development of DI exhibits a positive impact on the firm’s business performance [29] and organizational restructuring [32]. At the industry level, industrial upgrading and transformation are closely related to the gradual improvement of DI [34]. At the regional level, regions with developed DI have faster economic growth [31] and a better living environment [26].

As for the insurance industry, DI also influences it profoundly. Specifically, the more complete the DI, the wider the insurance company’s distribution channels, the stronger the risk management capabilities, and the better the quality and speed of insurance services [35]. Therefore, DI is an essential prerequisite for DTII.

The existing studies on the measurement of DI are diverse [36, 37]. In this paper, we follow the approach by taking the mobile phone penetration rate, the density of Internet access ports, the proportion of Broadband Internet users, and the proportion of mobile Internet users as the proxy variables for the DI [37].

### DP

DP refers to a technical framework that integrates efficient information collection, superb data analysis and processing, and open and diversified subjects in the era of the digital economy [27]. DP is characterized by flexibility, reduction of searching costs and transaction costs, and network effects [38]. In the DP, the complex and diverse interactions among different subjects, including enterprises, governments, and individuals, collectively shape the ecosystem of the DP. Currently, most scholars conduct research on DP from the dimensions of economics, information technology, organization, and strategy, and there are few studies involving insurance and DP [39, 40].

The construction and application of DP play a pivotal role in DTII [41]. Firstly, it enhances DTII by improving the external societal environment and the efficacy of government oversight. Secondly, from the insurance industry’s perspective, DP plays a vital role in amassing customer resources and data assets swiftly. It serves as a pivotal instrument for insurance companies’ digital transformation and contributes to building the insurance industry’s digital ecosystem, thus significantly expediting the sector’s digital evolution. Therefore, we try to measure DP from the following 3 perspectives: network society, e-government, and digital transformation of insurance companies:

*(1) Network society*. On the basis of electronic information technology, the network society consists of information resources as the basic development resources, the information service industry as the fundamental social industry, and digitalization and networking as the underlying social interaction mode [42]. With the continuous advancement of information technology, the development of a network society has caused changes in lifestyle and social structure.

Among them, e-commerce is the key product of the network society and acts a pivotal role in the output of insurance technology, the cross-industry integration in the insurance industry, and the construction of the insurance digital ecosystem [43]. In addition, different from the traditional industrial society, the social development capability presents the characteristics of networking and informatization in the network society, which also deserves our attention [44]. To sum up, we choose e-commerce capability and social development capability to measure NS.

*(2) E-Government*. As an important part of modern government administrative tools, e-government means that government agencies use information technology to promote public goals and improve public services, transforming their relationships with citizens, businesses, and government departments, namely G2C, G2B, and G2G [45]. The development of e-government breaks time and space constraints and has the function of improving efficiency and performance, especially in dealing with government-business relationships (i.e., e- G2B), The existing studies have proven that E-government facilitates innovation and performance of businesses and transformative development of industries [46]. Therefore, we use government websites usage to proxy E-government.

*(3) Degree of Digital Transformation in Insurance Companies (DDTIC)*. Digital transformation of insurance companies is a concrete manifestation of the application of DP in the insurance industry. At present, there is no unified definition for digital transformation of insurance companies. We try to define the DDTIC according to the definition of enterprise-level digital transformation. The current definition of enterprise digital transformation relies on the utilization of new digital technologies to transform the enterprise’s business, organizational structure, and business model [47, 48]. On this basis, we propose that the digital transformation of insurance companies is a dynamic process of using new digital technologies (such as big data, AI, distributed ledger technology, etc.) to empower the development of insurance companies, mainly in terms of reorganizing the company’s business and functions, as well as changing the company’s organizational structure and management system.

At present, most current research on measuring DDTIC uses case studies to evaluate specific insurance companies [49, 50]. These cases are concentrated on large insurance companies while there are fewer studies on small and medium-sized insurance companies. To reflect the degree of digital transformation of insurers more comprehensively, we conduct the widely accepted text analysis method [51, 52] by counting the frequency of keywords related to digital transformation from Insurance companies’ annual reports through the Python program.

### DA

The digital revolution has exerted huge influences on the development speed and quality of the insurance industry, and the application of digital technology has gradually become the main source of competitive advantage and efficiency in the insurance industry [53]. DA is the use of advanced digital technologies that can drive other technologies and generate new business, market, and business models [54].

Our research mainly focuses on the use and development of the above digital technology applications in the insurance industry. Specifically, we believe that DA of the insurance industry should be divided into the following two aspects. First, in a narrow sense, DA within the insurance industry primarily focuses on the industry’s internal aspects, exemplified by the growth of InsurTech. Secondly, in a broad sense, the scope of DA in the insurance sector extends to the wider financial industry [2]. As an important part of the financial industry, DA in the financial industry is also largely applicable to the insurance industry, especially the development of digital financial services (DFS).

*(1) InsurTech Development Index (IDI)*. InsurTech includes the integrated use of digital technologies to improve insurance ecology through channels such as product innovation, marketing, and enterprise management [8]. The application of InsurTech has innovated insurance sales channels, deepened insurance protection functions, and improved insurance service levels [21]. InsurTech is the concentration of DA in the insurance industry at this stage.

There are two main approaches to measuring the development of InsurTech. One is the textual analysis method mentioned in the previous section, which is based on the perspective of the supply side, i.e., insurance companies [14]. The other one is from the demand-side perspective, we use consumer microdata to measure the development of InsurTech [55]. Furthermore, this study constructs the IDI by using the insurance business subset under the depth of use in the China Digital Inclusive Finance Index compiled by the Institute of Digital Finance Peking University (IDFPU) (see https://idf.pku.edu.cn/zsbz/515313.htm). The index adopts the underlying data of transaction accounts of Ant Financial Services, and the number of insured users per 10,000 Alipay users, the number of insurance purchases per capita, and the insurance amount per capita are standardized processing. The weights of specific indicators are determined on basis of the hierarchical analysis method and the coefficient of variation method to form the IDI’s sub-index.

*(2) Digital Financial Services (DFS)*. Digital Financial Services (DFS) is a widely used digital technology that provides basic financial services to consumers [56]. The level of DFS reflects the development of financial inclusion in the context of the digital economy. Some scholars equate the DFS directly to digital financial inclusion because the essence of financial inclusion is to provide access to and use of formal financial services by all [57]. Others also see the DFS as a crucial component of digital financial inclusion because they are concerned with the accessibility of services by digital financial institutions [58, 59]. Our measurement criteria for DFS are based on the second point of view. Furthermore, we believe that the examination of the liquidity, affordability, and convenience of DFS shows it has advantages of low cost and low threshold. In terms of index selection, we select e-loan, electronic credit, and users’ QR code payments to evaluate DFS, and the data also comes from the IDFPU.

## Measurement

### Index system

We invite 14 experts to help us select indexes from different dimensions and analyze the importance of each index. 10 of them are from universities, 1 is from a large insurance company, 1 is from the government, and 2 of them work in research institutions. These experienced experts have long been concerned with the development and trends in the insurance industry, financial industry, and information engineering. Through the experts’ suggestions and relevant literature references [36, 51], we try to collect indicators from 3 dimensions: DI (U_1_), DP (U_2_), DA (U_3_), with 9 indicators (see Table 1). The procedure of selecting indicators follows three basic rules [60]. First, comprehensiveness, i.e., a complete indicator system should fully reflect the specifics of the sub-indicator layer. Second, feasibility, i.e., the selected indicators should be quantifiable and usable. Last but not the least, comparability, i.e., indicators of different levels can be compared with each other.

**Table 1:** Index system of DTII.

Target layer	Criteria layer	Index layer	Description	Objective weight	Subjective weight	Combined weight
Index system of DTII	Digital infrastructure (U_1_)	Mobile phone penetration rate (U_11_)	Number of telephones per 100 inhabitants	0.1114	0.0158	0.0466
		Density of Internet access ports (U_12_)	Number of Internet broadband access port (10,000) /Number of permanent Population (10,000)	0.1111	0.0626	0.0925
		Proportion of Broadband Internet Users (U_13_)	Number of Internet broadband access users (10,000) /Number of permanent population (10,000)	0.1111	0.0353	0.0694
		Proportion of Mobile Internet Users (U_14_)	Number of mobile internet users (10,000) /Number of permanent population (10,000)	0.1112	0.065	0.0946
	Digital platform (U_2_)	Network Society (U_21_)	Including: e-commerce index and social development index	0.1112	0.1697	0.1523
		E-government (U_22_)	Government website usage	0.1109	0.0435	0.077
		Degree of Digital Transformation in Insurance Companies (U_23_)	Frequency of key words related to digital transformation in insurance company annual reports	0.1111	0.0998	0.1167
	Digital applications (U_3_)	InsurTech Development Index (U_31_)	Including: number of insured users per 10,000 Alipay users, number of insurance purchases per capita, insurance amount per capita	0.1112	0.0935	0.1131
		Digital Financial Services (U_32_)	Including: e-loan, electronic credit, users’ QR code payment	0.1108	0.4148	0.2378

### Data sources

We collect a range of data for 31 provinces in China as study areas from 2014–2020 and finally select 9 basic indexes (Second-level indexes). Four indexes (U_11_- U_14_) come from the official website of the National Bureau of Statistics (see http://www.stats.gov.cn/). Four indexes (U_21_- U_22_) come from the China Information Society Development Report, released by the National Information Center (see http://www.sic.gov.cn/). The raw data for one index(U_23_) comes from annual reports of insurance companies disclosed by the Insurance Association of China (see http://icidp.iachina.cn/ICID/). Four indexes (U_31_- U_32_) come from the Peking University Digital Financial Inclusion Index of China (PKU_DFIIC) (see https://idf.pku.edu.cn/).

### Index analysis

#### Index standardization

All these indicators of different dimensions contain useful information in some aspects of DTII, however, if one indicator or indicators of the same dimension are used alone, it may lead to a one-sided interpretation of the current situation of DTII. We try to construct a comprehensive digital transformation index for the insurance industry. Before the index synthesis, we standardize the indicators with different natures and measurement units.

The standardization formula for positive indicators is:

$$Y_{ij}=\frac{X_{ij}-\min\left(X_{j}\right)}{\max\left(X_{j}\right)-\min\left(X_{j}\right)},(i=1,2,\ldots m;j=1,2,\ldots n)$$ (1)

The standardization formula of negative indicators is:

$$Y_{ij}=\frac{\max\left(X_{j}\right){-X}_{ij}}{\max\left(X_{j}\right)-\min\left(X_{j}\right)},(i=1,2,\ldots m;j=1,2,\ldots n)$$ (2)

where *X*_*ij*_ represents the j-th indicator value in the i-th province, min(*X*_*j*_) and max(*X*_*j*_) are the minimum and maximum values of the j-th indicator, respectively. *Y*_*ij*_ represents the indicator value after standardization. The indicators in this paper are all positive indicators, so we only use the standardization formula of positive indicators.

#### Weights choice

We assign weights to the indicators in terms of subjective and objective dimensions and then synthesize a composite weight. In particular, we use the entropy weight method (EWM) to judge objective weights and the analytic hierarchy process (AHP) method to generate subjective weights respectively. Finally, we use the minimum relative entropy method [61] to calculate combined weights.

Combining both qualitative and quantitative analysis approaches, the AHP method is a multi-objective decision analysis method aiming to decompose problems that are complex into several dimensions and several factors. Its logic is to compare the importance of two indicators by establishing a judgment matrix. After obtaining the maximum eigenvalues of the judgment matrix and the corresponding eigenvectors, we can derive the weights of the importance of different solutions or indicators. In addition, we invite 14 experts from insurance companies, universities, government, and research institutions to evaluate the importance of indicators for sake of the accuracy and objectivity of the judgment matrix.

In line with the AHP method, the importance of factors should be comparable at the same level. In the AHP questionnaire given to experts, indicators are divided into two layers for importance comparisons. The first tier is a two-by-two comparison between the three indicators: DI (U_1_), DP (U_2_), and DA(U_3_).

The second layer reflects the comparison between the indicators of each sub-criterion. For instance, the importance of four indicators in DI, i.e., the mobile phone penetration rate, the density of Internet access ports, the proportion of broadband Internet users, and the proportion of mobile Internet users (U_14_) are compared in pairs. We divide the importance measure into 9 levels from 1 as the equally important to 9 as the absolutely important.

In addition, given the subjective nature of this evaluation, it is essential to maintain logical coherence and consistency among experts. To achieve this, we conduct consistency tests on each expert’s pairwise comparison matrix to ensure that the consistency ratio is no larger than 0.10. The results confirm that all expert evaluation matrices pass the consistency test. With the AHP, we calculate the subjective weights ${w_{AHP}}_{j}$, *j* = 1, 2,…, 17.

As an objective weighting method, the EWM uses information entropy to compute the entropy weight of each index, then correct them according to each index to obtain a more objective index weight. The EWM is a complicated method, and the calculation process is as follows:

Step 1: Index standardization.

Step 2: Calculate the specific gravity P_*ij*_ of each indicator and determine the entropy value *H*_*j*_, as shown in Eqs (3) and (4).

$$P_{ij}=\frac{Y_{ij}}{\sum_{i=1}^{31}Y_{ij}},(i=1,2,\ldots 31;j=1,2,\ldots 9)$$ (3)

$$H_{j}=-\frac{1}{lnm}\sum_{i=1}^{m}P_{ij}\times lnP_{ij},(m=31)$$ (4)

Step 3: Calculate the entropy weight of the j-th indicator *W*_*j*_ and generate the composite score of j-th indicator *F*_*j*_, the process is as follows:

$$W_{{EWM}_{j}}=\frac{1-H_{j}}{\sum_{j=1}^{17}\left(1-H_{j}\right)},(j=1,2,\ldots 9)$$ (5)

$$F_{{EWM}_{j}}=\sum_{j=1}^{17}W_{{EWM}_{j}}\times Y_{ij},(j=1,2,\ldots 9)$$ (6)

*W*_*j*_ is a combined weight of $W_{{AHP}_{j}}$ and $W_{{EWM}_{j}}$, Obviously, the value of *W*_*j*_ should be as close as possible to $W_{{AHP}_{j}}$ and $W_{{EWM}_{j}}$. Referring to the minimum relative entropy method [61, 62], we calculate *W*_*j*_ as follows:

$$W_{j}=\frac{\sqrt{W_{{AHP}_{j}}-W_{{EWM}_{j}}}}{\sum_{j=1}^{17}\sqrt{W_{{AHP}_{j}}-W_{{EWM}_{j}}}},(j=1,2,\ldots 9)$$ (7)

In addition, after collecting and sorting out the valid expert questionnaires, we select the Expert Choice software to compute the subjective weights of each index, and we also use Stata 17.0 to obtain the objective weights and comprehensive weights. The specific weights of each index are shown in Table 1.

## Results

### Development trend

Based on the index system and index development methodology described above, we compile the comprehensive digital transformation indexes for the insurance industry in 31 provinces (including provinces, municipalities directly under the central government, and autonomous regions) in mainland China from 2014 to 2020. Moreover, we also construct Secondary indexes, including DI index, DP index, and DA index. In this section, we use some intuitive and rigorous statistical methods to briefly explain the development trend and spatial characteristics of DTII in China as reflected by this set of indexes.

### The development trend of DTII in China

**Fig 2:**
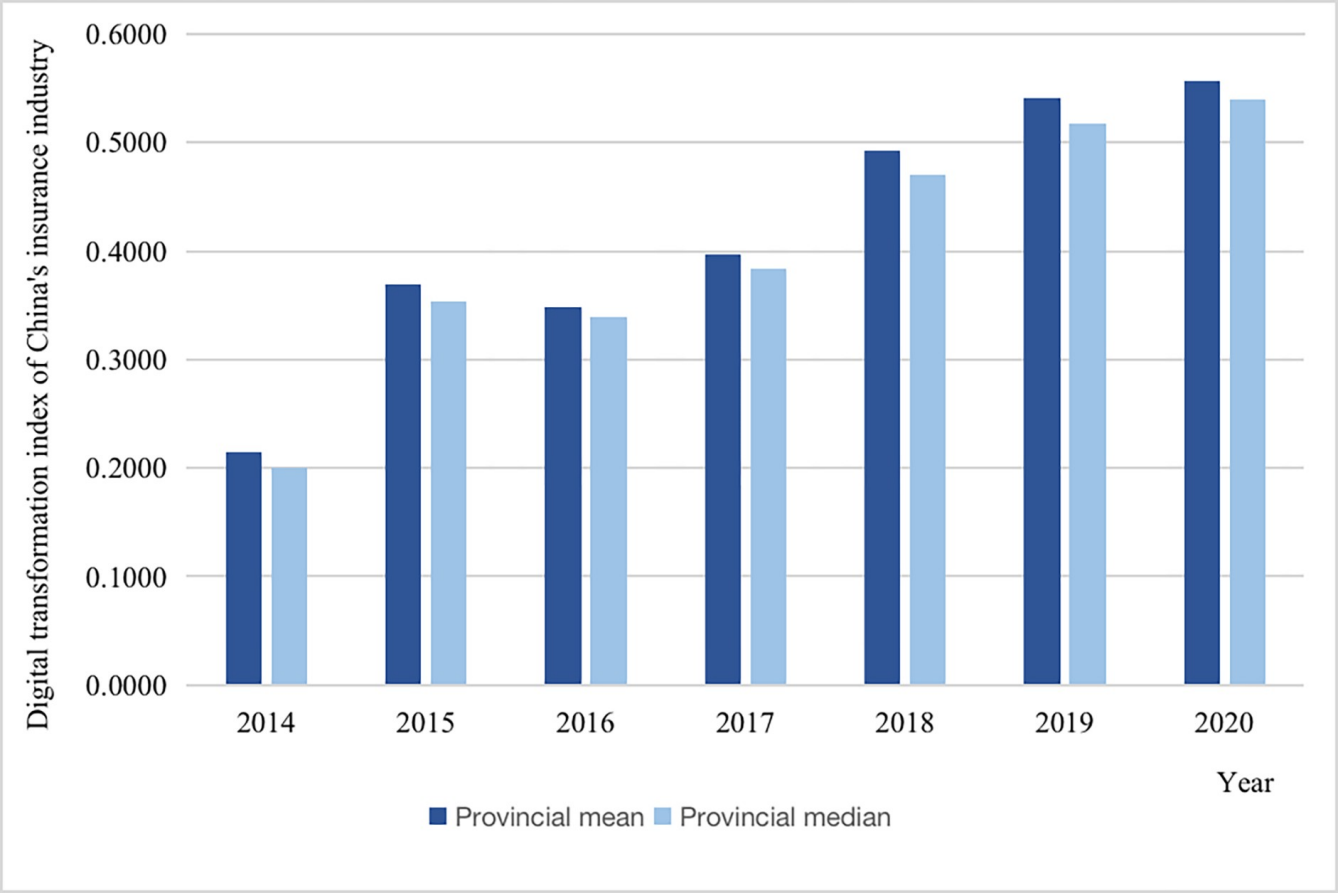
Mean and median values of DTII.

Fig 2 shows the annual mean and median of DTII in 31 provinces in mainland China from 2014 to 2020. We can see that DTII in China has made great progress during this period. Specifically, the median value of DTII in each province is 0.2 in 2014 and gradually rises to 0.54 in 2020, with an average annual growth rate of 20.46%, reflecting that DTII in China is advancing rapidly. From the regional perspective, the eastern regions, such as Beijing, Shanghai, and Zhejiang Province, have a higher digital transformation degree in the insurance industry (see S1 File). This aligns with the fact that the eastern regions in China have historically exhibited more advanced economic and social development. Thus, it is logical for them to possess advantages in promoting DTII, particularly in terms of human and physical capital.

We can further divide digital transformation process of the insurance industry into the following two periods: the rising and then falling period from 2014 to 2016 and the steady development period from 2016 to 2020.

We believe that these development trends are related to different stages of DTII. In the first stage, DTII relies on the information infrastructure, and vigorously develops the digitalization of the insurance business. From 2012 to 2015, the Chinese government issues a series of policies, such as “Broadband China” and “Internet Plus” Strategies, to improve China’s DI. In August 2013, the State Council has issued the Notice of the State Council on the Issuance of the “Broadband China” Strategy and Implementation Plan. Accordingly, the Chinese government will gradually promote the construction of broadband and other network infrastructure in batches. Up to now, the Ministry of Industry and Information Technology and the National Development and Reform Commission have selected 120 cities (clusters) as “Broadband China” demonstration sites in three batches in 2014, 2015 and 2016 (see http://www.gov.cn/zwgk/2013-08/17/content_2468348.htm). In July 2015, the State Council released its “Internet+” strategy, elevating the digitization of business to the national development level. The “Internet+” is the deep integration of Internet innovations with all areas of the economy and society, promoting technological progress, efficiency improvement and organizational change, enhancing the innovation and productivity of the real economy, and forming a new form of economic and social development with the Internet as the infrastructure and innovation factor more widely (see http://www.gov.cn/zhengce/content/2015-07/04/content_10002.htm). Digital insurance products and services are also constantly being innovated and promoted in this process [63]. However, with the gradual improvement of the DI, the application of digital technology in the insurance industry is facing problems such as single product functions and insufficient business model innovation and DTII is facing huge challenges.

The second stage is after 2016. In this period, DTII is characterized by platform digitization as the core. With the advancement and application of digital technologies, the ability of insurance companies to analyze and use data has been significantly improved. Coupled with the increase in the number of users in various scenarios, DP has become a new product and service carrier in the insurance industry, and DTII continues to develop.

**Fig 3:**
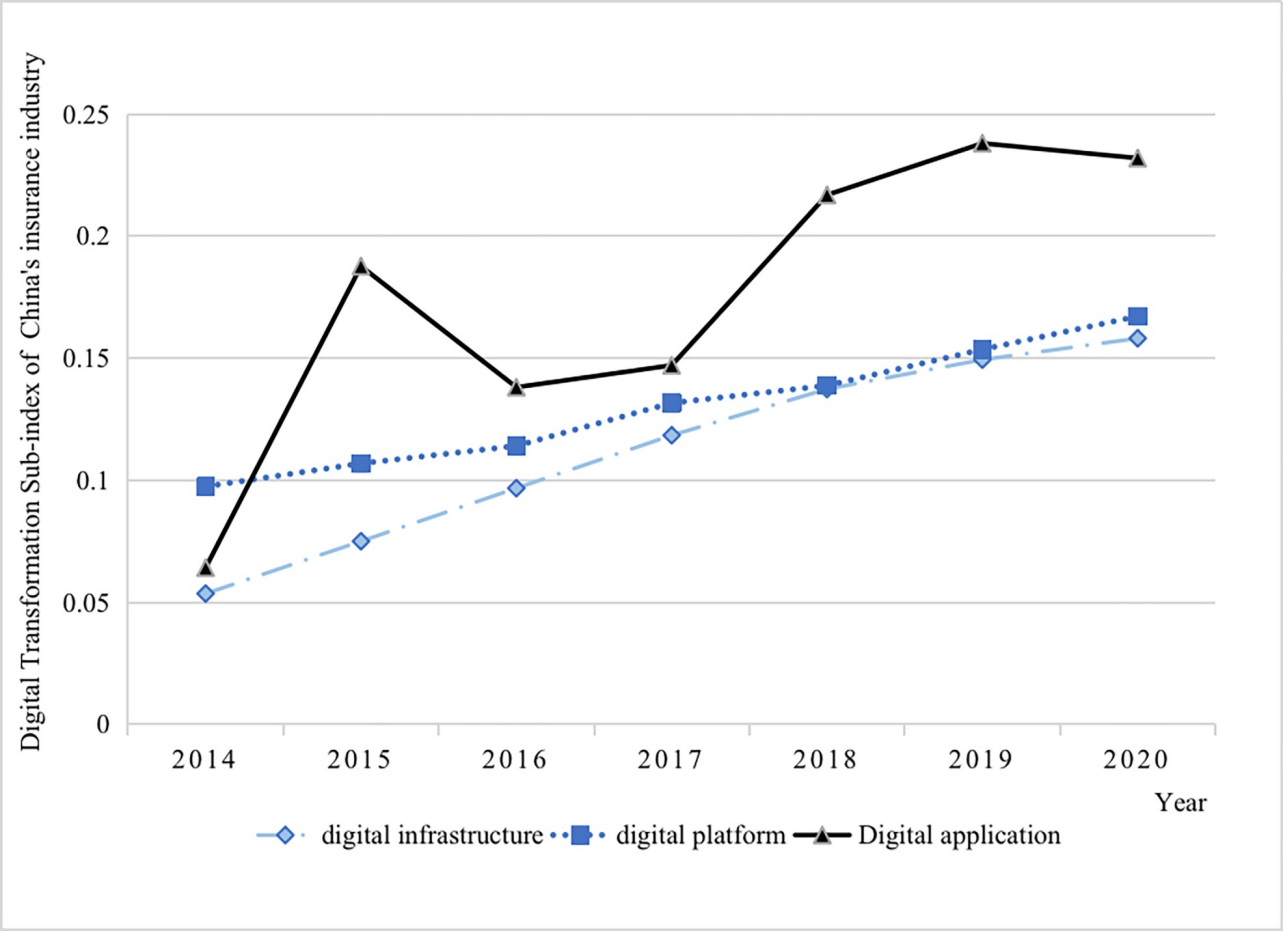
Secondary index of DTII.

The overall growth rate of the above insurance digital transformation index masks trends among secondary indicators of different dimensions (see Fig 3). In terms of sub-indexes, the DA index is the fastest growing and significantly higher than the other indicators, while the DP index has grown at the slowest speed during 2014 and 2020. Because of the continuous improvement of the DI, DA has gradually become an important driving force for DTII in various places. However, the construction of DP for the insurance industry is a continuous process of resource integration and structural reshaping, and its growth rate is relatively low.

### Regional convergence of DTII in China

With the rapid development of DTII, there are still some regional differences in DTII in China, just like most of China’s economic characteristics. Beijing, with the highest score in the insurance industry digital transformation index in 2020, is 2.3 times that of Tibet Province with the lowest score, but this difference is lower than the regional differences in traditional insurance. For example, in 2020, the per capita premium in Beijing, the highest, is 9.8 times that in Tibet Province, the lowest province. These phenomena indicate that DTII has better geographical penetration and creates a broader coverage compared with the development of the traditional insurance industry.

Moreover, we believe that the significance lies not only in the current smaller regional disparity in DTII compared to the traditional insurance industry but also in the potential for a continual reduction in this gap over time. If the regional differences in DTII can be gradually narrowed, even initially less-developed regions have the opportunity to bridge the gap and avoid being “lost at the starting line”, exemplifying the essence of DTII.

To demonstrate more rigorously the time trend of digital transformation development gap between regions in the insurance industry, we apply the regional economic convergence theory to discuss, and we mainly use the σ convergence model to verify [64]. The σ convergence is a description of the stock level, reflecting the difference and the dynamic process of this difference between regions. That is, if the difference is getting smaller, it can be assumed that there is convergence in digital transformation of the interregional insurance industry. Specifically, we calculate the *σ* convergence as follows:

$$\sigma_{t}=\sqrt{\frac{1}{n}\sum_{i=1}^{n}{\left({lnindex}_{it}-\frac{1}{n}\sum_{i=1}^{n}{lnindex}_{it}\right)}^{2}}$$ (8)

*i* represents region (province or municipality), *n* represents the number of regions, *t* represents year, *lnindex*_*it*_ represents the logarithmic value of DTII index in region *i* in year *t*, *σ*_*t*_ represents *σ* convergence test coefficients of DTII index in year *t*. If *σ*_*t*+1_<*σ*_*t*_, then it can be assumed that DTII in year *t*+1 is also more convergent than in year *t*.

**Fig 4:**
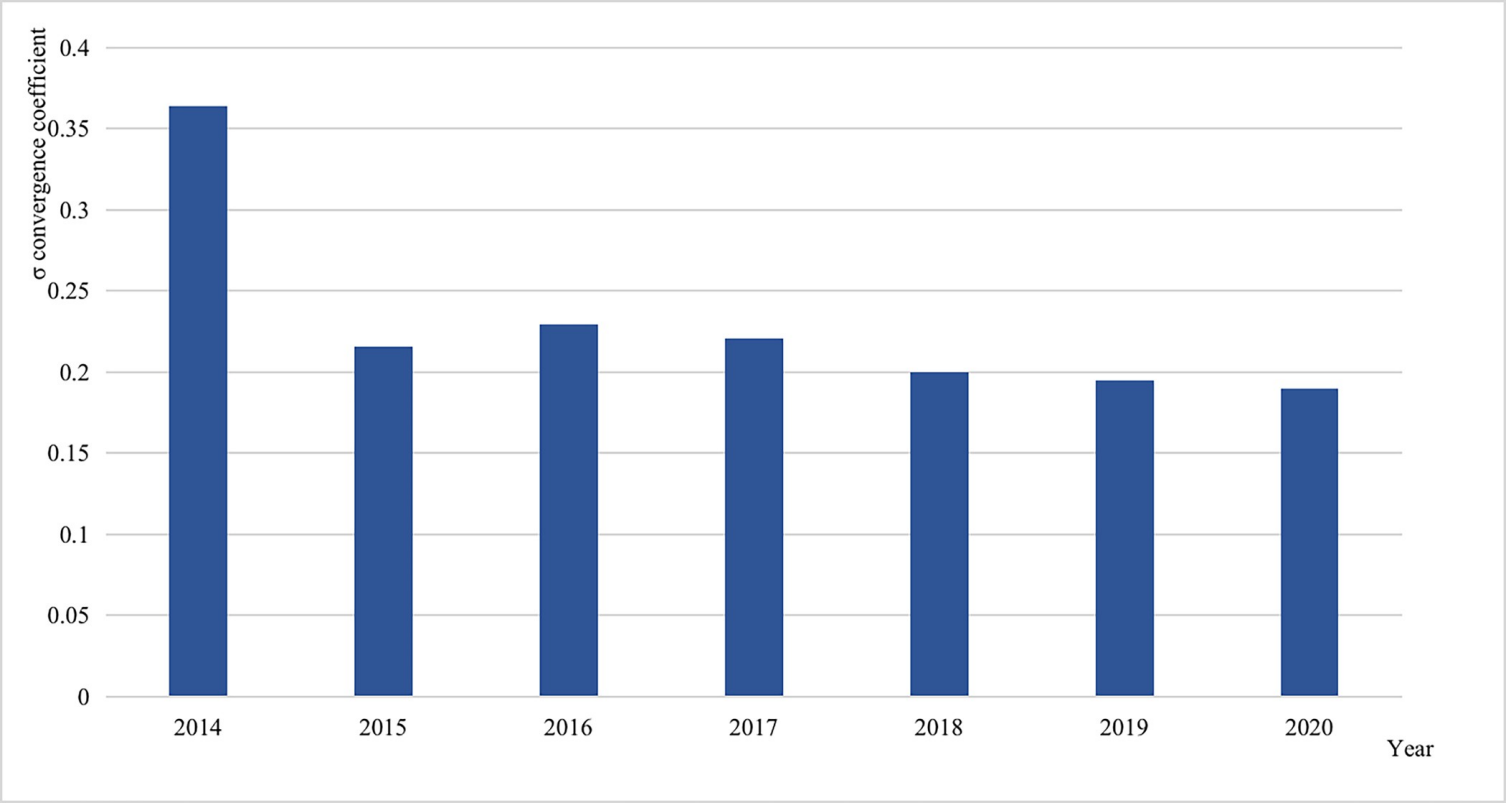
*σ* convergence coefficient of DTII.

In Fig 4, we report the provincial DTII indexes with yearly *σ* convergence coefficients from 2014 to 2020 separately. Overall, DTII has a convergence trend. Specifically, the *σ* convergence coefficient of digital transformation index of the Chinese provincial insurance industry declines from 0.37 in 2014 to 0.22 in 2015 but rebounds slightly in 2016. This indicates that the development of digital transition may play a very important role in alleviating the inadequacy and imbalance of the current development of the insurance industry in China.

## Spatial effects

In the previous section, we examine the convergence characteristics between regions in DTII, in this section, we will further analyze the spatial effects of insurance digital transformation, and answer the following questions: is there a spatial aggregation of DTII? Is there a spatial spillover effect between DTII and the development of the insurance industry?

### Spatial agglomeration

The study of spatial aggregation has been one of the hot topics recently. The current research results on DTII are rare, and we try to elaborate different views on the spatial aggregation of DTII based on the research related to economic growth transformation and digital transformation in the financial industry [65].

On the one hand, DTII can theoretically break through the traditional geographical and spatial limitations to achieve a low-cost and convenient allocation of insurance resources in distant regions. Therefore, the development of DTII in a certain region should not be significantly correlated with the geographical location of the region and the neighboring regions.

However, from another perspective, DTII, as a new business mode, still has to follow the basic laws of insurance industry development. DTII does not appear and develop out of nowhere, and its development still depends on status of the real economy and traditional insurance industry development. Moreover, DTII is highly related to some geographic factors for business diffusion [66]. Therefore, another reasonable hypothesis is that the development of DTII will still show a strong spatial agglomeration. Considering the above two considerations, apart from the convergence of digital transformation development in the insurance industry at the regional level, how to show and evolve its spatial agglomeration has become a valuable research question.

We use the Moran index to examine the spatial agglomeration of DTII, which ranges from -1 to 1. If its value is greater than 0, then there is positive spatial autocorrelation of digital transformation in the regional insurance industry, i.e., DTII has similar properties between neighboring regions, and provinces with a high degree of DTII and those with low development level cluster together. The larger the value, the stronger the positive autocorrelation of the spatial distribution as well as the agglomeration.

Furthermore, to examine the spatial dependence of specific regions beyond spatial autocorrelation, the local Moran index can be used. Local Moran analysis can provide spatial relationships between regions and neighboring regions. In local Moran analysis, the spatial relationship patterns of different regions are generally presented graphically. Specifically, by drawing a scatter plot of the local Moran index on a two-dimensional plane, DTII in each region is divided into cluster patterns in four quadrants into identify the spatial relationships between a region and its neighboring regions. We select the geographic distance matrix, i.e., the elements of the spatial weight matrix are the inverse of the squared distance between two regions.

**Fig 5:**
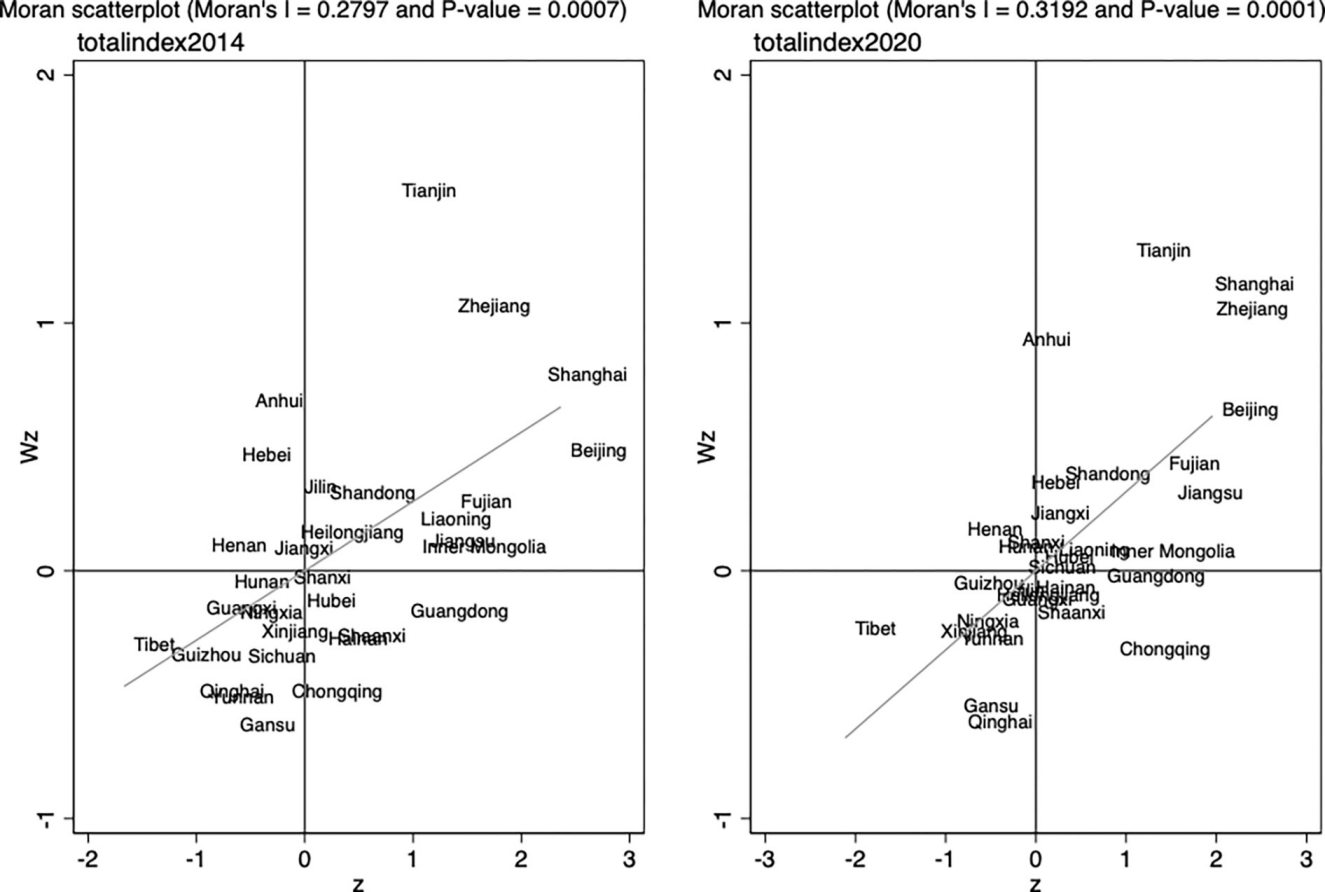
Local Moran index of DTII in 2014 and 2020.

Fig 5 shows a local Moran index scatter diagram of the provincial insurance industry digital transformation indexes in 2014 and 2020. We can see that most of the regions fall in quadrant 1 or quadrant 3, i.e. either high-high combination or low-low combination; only a few regions fall in quadrants 2 and 4. Most of the regions in quadrant 1 are the eastern coastal provinces and municipalities, with a high degree of digital transformation in their insurance industry and in the nearby areas; those in quadrant 3 belong to the central and western areas, where DTII in and around those regions is slow and low.

### Spatial spillover effect

With growing economic interconnections among regions, there is an observed tendency for interaction effects between them, giving rise to spatial autocorrelation. This phenomenon implies that the development of the insurance industry in one region may be influenced by developments in other regions. Meanwhile, on the basis of modern information networks as an important carrier, DTII takes digital knowledge and information as key production factors. Plus, with its characteristics of synergy, integration, and permeability, DTII can break through the limitation of geographical distance, go beyond the boundaries of space and region, achieve cross-regional division of labor and cooperation, and ultimately generate spatial spillover effects. Therefore, DTII in this region can also influence the development of insurance industry in other regions. To explain this phenomenon, we adopt a spatial measurement method to further discuss the relationship between DTII and the development of the insurance industry.

### Research design

*(1) Spatial dependence examination*. First, as in the Spatial Agglomeration section, we construct the spatial weight matrix. Secondly, we conduct the Moran index test. The results show that the Moran indexes from 2014 to 2020 pass the 10% significance test (Due to space constraints, we do not report this process in the manuscript), rejecting the original hypothesis of “no spatial autocorrelation”, suggesting that there is spatial autocorrelation and that the insurance industry development in this region is also influenced by the insurance industry development in neighboring regions.

*(2) Model Setting and variable selection*. Based on the spatial autocorrelation of insurance industry development and the fact that DTII exhibits a typical cross-regional division of labor and cooperation, we propose that the development of the insurance industry in one region is influenced not only by the development of the insurance industry in neighboring regions but also DTII in neighboring regions. Furthermore, according to LeSage and Pace’s [67] affirmation of the unbiasedness of spatial Durbin model (SDM) estimation, we construct the following SDM for spatial econometric analysis in Eq (9):

$${development}_{it}=\alpha +\rho W\;{development}_{it}+\beta {totalindex}_{it}+\eta W\;{totalindex}_{it}+\gamma X_{it}+\delta_{t}+\theta_{p}+\varepsilon_{it}$$ (9)

*i* represents the province, and *t* represents the year. The explained variable *development*_*it*_ represents the insurance industry development, and we take the logarithm of a premium income to measure it. The explanatory variable *totalindex*_*it*_ represents digital transformation index on the insurance industry. *W* represents the spatial weight matrix, and *W development*_*it*_ represents the spatial lag term of the insurance industry development, and *W totalindex*_*it*_ represents the spatial lag term of digital transformation index on the insurance industry.

In order to control the impact of various factors at the regional level on the research results, this study sets the following control variables *X*_*it*_ based on relevant literature research:

Economic development level (*GDP*). The current literature has demonstrated a positive correlation between the economic development level and the development of the insurance industry [2]. This study proxy for economic development level with the logarithm of GDP.

Population size (*Population*). The size of the local population is one of the key factors influencing the development of the insurance industry [68]. Our study employs the logarithm of the year-end resident population at the provincial level to measure population size.

Government governance (*Governance*). This variable affects the development of the insurance industry in terms of supervision, risk management, market promotion and social security etc. [69] Based on the collection of Chinese government work reports from 2014–2020 in each province, we follow the methodology of Lu et al. [70] and use text analysis to measure the governance level of regional governments.

The number of insurance company headquarters (*Number*). The number of insurance company headquarters can also have a significant impact on the development of the insurance industry [71]. This research measures this variable using the total number of insurance company headquarters in each province.

In addition, some unobservable factors also affect the insurance industry development, such as cyclical fluctuations in the economy, supply and demand shocks due to unexpected events, and the introduction of macroeconomic policies. There is no doubt that some of these changes are unobservable factors that vary with time but not with regions (provinces), so we include time-fixed effects *δ*_*t*_ in the model to control the changes in the overall economic environment. At the same time, some unobservable factors do not vary over time and could differ across regions (provinces), such as geographic location, economic structure, and regional culture. To avoid the biased estimation caused by such unobserved factors, province fixed effects *θ*_*p*_ are included in the model. The random disturbance term is denoted by *ε*_*it*_.

### Empirical results

*(1) Data description*. We conduct our research on DTII in China from 2014 to 2020. Data sources contain the National Bureau of Statistics, statistical yearbooks, insurance yearbooks, and government work reports, and we use Stata 17.0 measurement software to calculate the basic characteristic values of each variable (see Table 2).

**Table 2:** Basic characteristic values of variables.

Variable	Obs	Mean	Standard deviation	Min	Max
Development	217	11.1258	1.0674	7.1511	12.9475
Total index	217	0.3413	0.1265	0.0513	0.6628
GDP	217	10.9355	0.4002	10.1887	12.0130
Population	217	10.4435	0.8432	8.0864	11.7459
Governance	217	341.3594	72.3926	189	547
Number	217	5.8848	12.0923	0	54
Year	217	2017	2.0046	2014	2020
Region	217	1.0650	0.8420	0	2

Note: we use the premium income as a proxy variable of development in millions of dollars. The unit of GDP is yuan, and unit of FDI is 10 thousand dollars. The region variable is used as a categorical variable and takes the values of 0, 1, and 2, representing the West, Middle and East regions in China, respectively.

According to the data in Table 2, the mean value of insurance industry development is 11.1258, the standard deviation is 1.0674, and the maximum and minimum values are 7.1511 and 12.9475, respectively. The level of insurance industry development varies greatly among provinces. The mean value of DTII is 0.3413 and the minimum value is 0.0513, indicating that some provinces and cities have a low level of digital transformation in specific years.

*(2) Regression results*. Table 3 shows results of the spillover effects of insurance industry development, using the SDM based on LeSage and Pace’s [67] method. In particular, we estimate the spatial effects of DTII on the insurance industry development gradually.

**Table 3:** Results of spatial estimation.

Variable	Coefficient	Z value	Coefficient	Z value	Coefficient	Z value	Coefficient	Z value
	SDM		SDM		SAR		SAR	
	(1)		(2)		(3)		(4)	
Total index	6.7456***	15.87	3.5627***	18.68	5.8427***	14.17	3.5436***	20.82
W×Total index	-41352.17***	-5.21	-13571.82***	-2.91				
GDP			0.00713**	1.52			0.0685***	1.44
W×GDP			342.6266	0.47				
Population			0.8512***	32.71			0.8404***	34.09
W×Population			-2269.596**	-2.17				
Governance			0.0003*	1.36			0.0002***	1.97
W×Governance			-12.4222*	-1.78				
Number			0.0011*	1.77			0.0009**	2.99
W×Number			-60.1271*	-1.4				
N	217		217		217		217	
R^2^	0.1586		0.1205		0.5156		0.8727	
Time fixed effect	YES		YES		YES		YES	
Provincial fixed effect	YES		YES		YES		YES	
Log Likelihood	-216.2700		-3.6433		-228.9735		-9.7667	

Note

***, **, and * indicate significance at the level of 1%, 5%, and 10%, respectively.

We can extract two important pieces of information from the basic regression results in Column (1). First, the coefficient of the total index is significantly positive, which means that DTII in one region has a positive impact on the development of regional insurance industry as we expect.

The second and most interesting point is the coefficient of the cross-product term of the spatial weight matrix and the total index is significantly negative, indicating that DTII in one region is detrimental to that in its surrounding areas. In explaining this phenomenon, we try to use the “excessive competition” theory and the excessive competition among enterprises within the insurance industry from the following two aspects [72].

(i) Excessive competition in the product market. In recent years, there are a growing amount of insurance companies participating in the wave of digital transformation. Companies usually implement low-price competition strategies to seize market space and potential, which compresses the profit margins of companies, reduces R&D investment for product innovation, and lowers the probability of successful R&D, leading to ineffective development.

(ii) Excessive competition in the labor market. The clustering of insurance companies in a limited area triggers a massive increase in human capital demand, and large companies tend to be able to attract more and higher quality talent with their resource advantages in digital transformation process. The above analysis shows that DTII further deepens the excessive competition among insurance companies, which inhibits product innovation and the balanced development of human capital and thus is detrimental to the overall development of the insurance industry.

Besides, we add control variables gradually in Column (2) and we can see that although the coefficients of the total index and W*Total index decrease, they still significantly affect the development of the insurance industry in itself and surrounding areas. We also conduct a spatial analysis by using the Spatial Autoregressive Model in Column (3) and (4) the results support the conclusions we mention above.

## Discussion

We systematically analyze the construction of the evaluation system for DTII and the spatial effects of DTII in previous sectors. In this chapter, we will further discuss the spatial effects of DTII from the perspective of heterogeneity, and we focus on these questions: Does the spatial spillover effect of DTII have the same impact on property insurance companies as life insurance companies? Is the spatial spillover effect of DTII uniform across the eastern, central, and western regions?

### Product difference

We comparatively analyze the spatial spillover effects of property insurance companies and life insurance companies according to their operating products. Table 4 shows the results of subsample regressions, where column (1) is for the property insurance industry and column (2) is for the life insurance industry.

**Table 4:** Heterogeneity analysis: Product.

Variable	(1) *Property*		(2) *Life*	
	Coefficient	Z value	Coefficient	Z value
Total index	2.1717***	13.30	3.6874***	14.48
W×Total index	-10570.94***	-3.32	-23500.43***	-4.74
GDP	-.0115	-0.27	0.0160	0.25
W×GDP	-774.1615	-1.29	3306.65***	3.41
Population	0.8509***	41.00	1.0798***	33.56
W×Population	-336.7488	-0.31	-3444.484***	-2.85
Governance	0.0003	1.11	0.0006	1.60
W×Governance	-15.8584***	-2.58	-19.2434**	-2.02
Number	-0.0007	-0.52	0.0021	1.06
W×Number	-39.9450	-1.06	-93.7440	-1.59
Time fixed effect	YES		YES	
Provincial fixed effect	YES		YES	
N	217		217	
R^2^	0.1726		0.4497	
Log Likelihood	23.0870		-71.6666	

Note

***, **, and * indicate significance at the level of 1%, 5%, and 10%, respectively.

Specifically, the impact of DTII is different for the property insurance industry and the life insurance industry. Compared with the property insurance industry, digital transformation has a more obvious incentive influence on the development of the local life insurance industry and threatens the development of the surrounding life insurance industry. This may be because the property insurance industry and the life insurance industry are at different stages of development. Different from the vigorous blossom of the property insurance industry, the life insurance industry is at the end of the cyclical expansion, where the homogenization of life insurance products is serious. It is urgent to develop new products through digital transformation, stimulate new business models, and promote the sustainable development of the industry in the future. Therefore, digital transformation shows a more powerful effect on the development of the local life insurance industry.

In terms of the spatial spillover effect, as the life insurance market tends to be saturated, and with the continuous deepening of the application of digital transformation in the life insurance industry, the competition between life insurance companies is intensified and even excessive to seize the market and obtain more profits. Therefore, the digital transformation of the life insurance industry in some regions is against the development of the surrounding life insurance industry.

## Regional difference

We also analyze the spatial spillover effects of DTII in the Eastern, Middle, and Western regions, and the results are shown in Table 5. Among the impacts on the development of the insurance industry in these regions, the degree of digital transformation plays the largest role in the east, followed by the west, and the lowest when the middle.

**Table 5:** Heterogeneity analysis: Region.

Variable	(1) *East*		(2) *Middle*		(3) *West*	
	Coefficient	Z value	Coefficient	Z value	Coefficient	Z value
Total index	3.6733***	9.66	2.4535***	7.32	3.2687***	11.87
W×Total index	-151962.4***	-3.82	-112981.2**	-2.13	-22780.99*	-1.73
GDP	0.2901**	1.96	-0.9369***	12.85	-0.5393***	-5.22
W×GDP	56312.08***	3.22	67724.92***	4.26	3883.425***	2.82
Population	0.8249***	15.99	0.9369***	12.85	0.7148***	17.23
W×Population	-18080.61	-0.98	-72010.32***	-3.45	-7046.27*	-1.72
Governance	-.0002	-0.27	0.0005	1.51	0.0004	-1.09
W×Governance	17.1417	0.30	25.72258	0.46	-3.1534	-0.21
Number	-.0048	-1.47	0.0008	0.65	-0.0024	-0.76
W×Number	-478.7851	-0.87	-271.2811 **	-2.04	594.5977	1.30
N	77		56		77	
R^2^	0.0339		0.6227		0.0166	
Log Likelihood	-6.2014		32.4728		14.1289	

Note

***, ** and *indicate significance at the level of 1%, 5% and 10% respectively.

The results show that in eastern regions, DTII affects the development of the insurance industry more significantly, which is closely related to the prosperous economic development foundation of the eastern regions. We try to explain this result in the western regions from the policy incentive perspective.

The likely explanation is the recent surge in government efforts to enhance digital-related infrastructure in the western regions. As a result, DTII has shown improvements in the western region, contributing to the overall advancement of the insurance industry.

## Conclusions

This study defines the concept of DTII from three dimensions including DI, DP, and DA. Then we compile the insurance industry digital transformation index system of 31 provinces (including municipalities and autonomous regions) in mainland China from 2014 to 2020. Based on the indicator system of DTII, we briefly discuss the development trend, spatial characteristics, and spatial effects of DTII in China, and reach the following conclusions:

First, DTII in China has achieved leapfrog development from 2014 to 2020, and the growth of DA has gradually become an important driver for the development of DTII. The focus of DTII in China has shifted from business digitization to platform digitization, and the development model has shifted from quantitative expansion to qualitative growth.

Secondly, DTII in China shows strong regional convergence. On the whole, the gap between DTII in different regions is narrowed significantly. Digital transformation provides the possibility for underdeveloped regions of the insurance industry to realize digital transformation and catch up.

Thirdly, the spatial agglomeration of DTII in China is mainly concentrated in the eastern developed regions, while the agglomeration of DTII in the middle and western regions is relatively weak, reflecting the uneven development among regions for DTII in China.

Finally, there is a spatial spillover effect of DTII. Specifically, DTII has a positive effect on the development of the insurance industry in one region, while it is unfavorable to the development of the insurance industry in the neighboring regions, especially in the life insurance industry and the eastern regions.

This study contributes to a deeper understanding of DTII and its impact on insurance industry development. In the view of this, we further propose the following policy recommendations to foster the collaborative development of DTII and the insurance sector.

First, support the development of DA. Given the increasing importance of DA as a driver for DTII, policymakers should encourage and promote the application of digital technologies and tools within the insurance sector. This could include incentives, subsidies, etc. to help insurance companies embrace the digital revolution effectively.

Second, promote DP. As the focus of China’s DTII shifts to platform digitization, authorities should cultivate the development and utilization of DP. Specifically, the government should support the creation of insurance-related DP, facilitate collaboration between traditional insurers and InsurTech startups, and actively stimulate the sustainable development of a digital insurance ecosystem.

Third, Balanced regional development. To further narrow regional gaps and allow less developed regions to benefit from digital transformation, targeted policies should be formulated. These initiatives may involve investments in DI, establishing digital training and education programs, and providing incentives to attract digital insurers to undeveloped regions.

Finally, mitigate negative spatial spillover effects: In areas where DTII spatial spillover effects are unfavorable, especially the life insurance industry and the eastern region, specific policies, such as regulatory encouragement, strategic investments, etc. should be adopted to mitigate these adverse effects. Moreover, governments should conduct ongoing assessments and regulation of DTII, making necessary adjustments and refinements based on the evolving landscape of DTII and its impact on the insurance industry.

Our study has the following limitations: First, we select only a limited number of indicators to construct DTII indicator system because of the small amount of publicly available data on the insurance industry. The indicators we select may not be comprehensive. Secondly, we use the provincial data and ignore the intra-provincial differences. In the future study, we will collect city-level data for a more detailed analysis of DTII index. Thirdly, we do not conduct mechanism analysis in the spatial effect study of DTII, and we will examine its influencing factors and paths in future studies to improve the effect study of DTII.

## Supporting information

S1 File The word file *‘S1’* shows the top 10 regions of DTII Index from 2014 to 2020. (DOCX)

S1 Data Code and data for this study can be found in file *‘data&code*.*zip’*. Specifically, The file *‘code’* contains instructions for the descriptive statistical analysis and regression analysis in this study. File labeled *‘regression data*.*dta’* serves as the primary dataset for benchmark regression, while *‘Spatial regression*.*dta’* contains the foundational dataset for spatial regression. Additionally, *‘w*.*dta’* represents the spatial distance matrix utilized in this research.
(ZIP)
