# Supplementary material for: Index construction and application of digital transformation in the insurance industry: Evidence from China
Source: PLoS One. 2024 Jan 18;19(1):e0296899. doi: 10.1371/journal.pone.0296899 (PMC10796006; doi:10.1371/journal.pone.0296899)
Supplement: S1 File — (DOCX) [file pone.0296899.s001.docx]

**S1 Table. Top 10 Provinces and Municipalities in the digital transformation index on the insurance industry.**

| Year | Ranking | Province | Total index | Digital  Infrastucture  Index | Digital  Platform  Index | Digital  Appilcation  Index |
| --- | --- | --- | --- | --- | --- | --- |
| 2014 | 1 | Beijing | 0.4024 | 0.1649 | 0.1697 | 0.0679 |
| 2014 | 2 | Shanghai | 0.3858 | 0.1339 | 0.1663 | 0.0855 |
| 2014 | 3 | Fujian | 0.3221 | 0.0936 | 0.1659 | 0.0626 |
| 2014 | 4 | Zhejiang | 0.3203 | 0.1127 | 0.1402 | 0.0674 |
| 2014 | 5 | Jiangsu | 0.3000 | 0.0839 | 0.1465 | 0.0696 |
| 2014 | 6 | Inner Mongolia | 0.2940 | 0.0574 | 0.1429 | 0.0936 |
| 2014 | 7 | Liaoning | 0.2928 | 0.0825 | 0.1243 | 0.0861 |
| 2014 | 8 | Guangdong | 0.2855 | 0.0947 | 0.1333 | 0.0575 |
| 2014 | 9 | Tianjin | 0.2792 | 0.0452 | 0.1591 | 0.0749 |
| 2014 | 10 | Shaanxi | 0.2325 | 0.0545 | 0.1094 | 0.0687 |
| 2015 | 1 | Beijing | 0.5685 | 0.1947 | 0.1923 | 0.1816 |
| 2015 | 2 | Zhejiang | 0.5061 | 0.1722 | 0.1495 | 0.1844 |
| 2015 | 3 | Shanghai | 0.4976 | 0.1377 | 0.1742 | 0.1857 |
| 2015 | 4 | Fujian | 0.4754 | 0.1184 | 0.1755 | 0.1815 |
| 2015 | 5 | Inner Mongolia | 0.4718 | 0.0699 | 0.1683 | 0.2335 |
| 2015 | 6 | Jiangsu | 0.4552 | 0.1192 | 0.1559 | 0.1802 |
| 2015 | 7 | Liaoning | 0.4507 | 0.1054 | 0.1346 | 0.2107 |
| 2015 | 8 | Tianjin | 0.4395 | 0.0611 | 0.1846 | 0.1938 |
| 2015 | 9 | Hainan | 0.4277 | 0.0815 | 0.1094 | 0.2369 |
| 2015 | 10 | Guangdong | 0.4071 | 0.1122 | 0.1377 | 0.1573 |
| 2016 | 1 | Beijing | 0.5465 | 0.2104 | 0.1883 | 0.1478 |
| 2016 | 2 | Inner Mongolia | 0.4849 | 0.0922 | 0.1865 | 0.2063 |
| 2016 | 3 | Zhejiang | 0.4758 | 0.1802 | 0.1622 | 0.1334 |
| 2016 | 4 | Shanghai | 0.4560 | 0.1504 | 0.1697 | 0.1360 |
| 2016 | 5 | Jiangsu | 0.4537 | 0.1436 | 0.1705 | 0.1395 |
| 2016 | 6 | Fujian | 0.4414 | 0.1286 | 0.1888 | 0.1240 |
| 2016 | 7 | Tianjin | 0.4246 | 0.0938 | 0.1778 | 0.1530 |
| 2016 | 8 | Liaoning | 0.4131 | 0.1300 | 0.1425 | 0.1406 |
| 2016 | 9 | Shaanxi | 0.3787 | 0.1063 | 0.1335 | 0.1389 |
| 2016 | 10 | Guangdong | 0.3786 | 0.1286 | 0.1468 | 0.1033 |
| 2017 | 1 | Beijing | 0.6168 | 0.2424 | 0.2038 | 0.1706 |
| 2017 | 2 | Zhejiang | 0.5749 | 0.2105 | 0.1882 | 0.1762 |
| 2017 | 3 | Shanghai | 0.5478 | 0.1821 | 0.1787 | 0.1870 |
| 2017 | 4 | Jiangsu | 0.5366 | 0.1778 | 0.1946 | 0.1641 |
| 2017 | 5 | Fujian | 0.5176 | 0.1512 | 0.2099 | 0.1565 |
| 2017 | 6 | Inner Mongolia | 0.4963 | 0.1149 | 0.2236 | 0.1578 |
| 2017 | 7 | Tianjin | 0.4674 | 0.1189 | 0.1901 | 0.1584 |
| 2017 | 8 | Guangdong | 0.4550 | 0.1451 | 0.1682 | 0.1417 |
| 2017 | 9 | Liaoning | 0.4378 | 0.1392 | 0.1562 | 0.1423 |
| 2017 | 10 | Shandong | 0.4121 | 0.1160 | 0.1491 | 0.1471 |
| 2018 | 1 | Beijing | 0.7184 | 0.2331 | 0.2126 | 0.2727 |
| 2018 | 2 | Zhejiang | 0.7065 | 0.2197 | 0.1973 | 0.2896 |
| 2018 | 3 | Shanghai | 0.6801 | 0.1889 | 0.1798 | 0.3113 |
| 2018 | 4 | Fujian | 0.6563 | 0.1770 | 0.2192 | 0.2600 |
| 2018 | 5 | Jiangsu | 0.6500 | 0.1863 | 0.2056 | 0.2581 |
| 2018 | 6 | Guangdong | 0.5910 | 0.1679 | 0.1739 | 0.2492 |
| 2018 | 7 | Tianjin | 0.5782 | 0.1463 | 0.1995 | 0.2324 |
| 2018 | 8 | Inner Mongolia | 0.5393 | 0.1348 | 0.2371 | 0.1673 |
| 2018 | 9 | Liaoning | 0.5207 | 0.1472 | 0.1650 | 0.2086 |
| 2018 | 10 | Shandong | 0.5089 | 0.1300 | 0.1556 | 0.2233 |
| 2019 | 1 | Zhejiang | 0.7645 | 0.2283 | 0.2171 | 0.3191 |
| 2019 | 2 | Beijing | 0.7601 | 0.2376 | 0.2188 | 0.3037 |
| 2019 | 3 | Shanghai | 0.7429 | 0.2121 | 0.1823 | 0.3485 |
| 2019 | 4 | Jiangsu | 0.7051 | 0.1969 | 0.2254 | 0.2828 |
| 2019 | 5 | Fujian | 0.7009 | 0.1844 | 0.2367 | 0.2799 |
| 2019 | 6 | Guangdong | 0.6417 | 0.1709 | 0.1894 | 0.2815 |
| 2019 | 7 | Tianjin | 0.6391 | 0.1779 | 0.2022 | 0.2590 |
| 2019 | 8 | Inner Mongolia | 0.6046 | 0.1417 | 0.2686 | 0.1943 |
| 2019 | 9 | Chongqing | 0.6026 | 0.1663 | 0.1948 | 0.2416 |
| 2019 | 10 | Shandong | 0.5622 | 0.1446 | 0.1726 | 0.2450 |
| 2020 | 1 | Beijing | 0.7672 | 0.2402 | 0.2309 | 0.2960 |
| 2020 | 2 | Zhejiang | 0.7602 | 0.2187 | 0.2350 | 0.3065 |
| 2020 | 3 | Shanghai | 0.7585 | 0.2328 | 0.1874 | 0.3383 |
| 2020 | 4 | Jiangsu | 0.7149 | 0.1964 | 0.2435 | 0.2750 |
| 2020 | 5 | Fujian | 0.7037 | 0.1881 | 0.2526 | 0.2630 |
| 2020 | 6 | Tianjin | 0.6653 | 0.1987 | 0.2122 | 0.2544 |
| 2020 | 7 | Chongqing | 0.6450 | 0.1705 | 0.2359 | 0.2386 |
| 2020 | 8 | Inner Mongolia | 0.6348 | 0.1498 | 0.2957 | 0.1894 |
| 2020 | 9 | Guangdong | 0.6304 | 0.1637 | 0.2037 | 0.2630 |
| 2020 | 10 | Shandong | 0.5803 | 0.1462 | 0.1861 | 0.2481 |
